# Concurrent *Zrsr2* mutation and *Tet2* loss promote myelodysplastic neoplasm in mice

**DOI:** 10.1038/s41375-022-01674-2

**Published:** 2022-08-27

**Authors:** Cristian Garcia-Ruiz, Cristina Martínez-Valiente, Lourdes Cordón, Alessandro Liquori, Raúl Fernández-González, Eva Pericuesta, Juan Sandoval, José Cervera, Alfonso Gutiérrez-Adán, Alejandra Sanjuan-Pla

**Affiliations:** 1 Hematology Research Group, Instituto de Investigación Sanitaria La Fe (IISLAFE), Avda. Fernando Abril Martorell, 106, 46026 Valencia, Spain; 2 Centro de Investigación Biomédica en Red de Cáncer (CIBER-ONC), Av. Monforte de Lemos, 3-5. Pabellón 11. Planta 0, 28029 Madrid, Spain; 3 Animal Reproduction Department, INIA-CSIC, Ctra. de La Coruña, km 5,9, 28040 Madrid, Spain; 4 Epigenomics Core Facility, Instituto de Investigación Sanitaria La Fe (IISLAFE), Avda Fernando Abril Martorell, 46026 Valencia, Spain; 5 Hematology Service, Hospital Universitario y Politécnico La Fe, Avda. Fernando Abril Martorell, 106, 46026 Valencia, Spain; 6 Genetics Unit, Hospital Universitario y Politécnico La Fe, Avda. Fernando Abril Martorell, 106, 46026 Valencia, Spain

**Keywords:** Myelodysplastic syndrome, Haematopoietic stem cells, Cancer genetics

## Abstract

RNA splicing and epigenetic gene mutations are the most frequent genetic lesions found in patients with myelodysplastic neoplasm (MDS). About 25% of patients present concomitant mutations in such pathways, suggesting a cooperative role in MDS pathogenesis. Importantly, mutations in the splicing factor *ZRSR2* frequently associate with alterations in the epigenetic regulator *TET2*. However, the impact of these concurrent mutations in hematopoiesis and MDS remains unclear. Using CRISPR/Cas9 genetically engineered mice, we demonstrate that *Zrsr2*^m/m^*Tet2*^−/−^ promote MDS with reduced penetrance. Animals presented peripheral blood cytopenia, splenomegaly, extramedullary hematopoiesis, and multi-lineage dysplasia, signs consistent with MDS. We identified a myelo-erythroid differentiation block accompanied by an expansion of LT-HSC and MPP2 progenitors. Transplanted animals presented a similar phenotype, thus indicating that alterations were cell-autonomous. Whole-transcriptome analysis in HSPC revealed key alterations in ribosome, inflammation, and migration/motility processes. Moreover, we found the MAPK pathway as the most affected target by mRNA aberrant splicing. Collectively, this study shows that concomitant *Zrsr2* mutation and *Tet2* loss are sufficient to initiate MDS in mice. Understanding this mechanistic interplay will be crucial for the identification of novel therapeutic targets in the spliceosome/epigenetic MDS subgroup.

## Introduction

Genetic mutations in splicing factors are observed in circa 50% of human myelodysplastic neoplasm (MDS) and about 45% of cases carry mutations in epigenetic regulators. Importantly, a subgroup of patients (25%) presents mutations in both splicing factors and epigenetic regulators, indicating possible cooperation among both functional categories in the pathomechanism of the disease.

The splicing factor *ZRSR2* is mutated in about 5% of patients with MDS and appears associated with mutations in the epigenetic regulator *TET2* [1]. ZRSR2 is implicated in the minor spliceosome and acts on U2-type introns (99.7% of introns in the human genome) as well as on U12-type introns (only present in approximately <0.3% of introns of all human genes) [2]. Mutations in *ZRSR2* are very diverse and comprise nonsense, frameshift, missense, and splice-site mutations. While different hotspots are affected in other mutated splicing factors, mutations in *ZRSR2* distribute across the entire coding region leading to protein loss-of-function [3]. *ZRSR2* mutations affect predominantly males and cause abnormal splicing via intron retention of U12-dependent introns [4–7]. Apart from MDS, *ZRSR2* mutations have also been reported in other hematological malignancies including chronic myelomonocytic leukemia (CMML) [3], myeloproliferative neoplasms (MPN) [3], and blastic plasmacytoid dendritic cell neoplasm (BPCDN) [8]. Functionally, shRNA-mediated *ZRSR2* silencing in AML cell lines induced aberrant retention of U12-type introns, diminished cell growth, and altered myeloid differentiation [4]. Recently, two in vivo *Zrsr2* knockout mouse models have been developed. Constitutive germline deletion of *Zrsr2* in mice demonstrated that *Zrsr2* is dispensable for hematopoietic development and that absence of a major phenotype is due to a *Zrsr1* compensatory mechanism [6]. In contrast, *Zrsr2* hematopoietic conditional knockout mice resulted in enhanced hematopoietic stem cell (HSC) self-renewal in vitro and in vivo [7].

On the other hand, TET2 promotes DNA demethylation by oxidizing the methyl group to 5-hydroxymethylcytosine. *TET2* is the most frequently mutated gene in MDS (25–35%) [9], and biallelic *TET2* gene inactivation is frequently observed in myeloid neoplasms [10]. In addition, *TET2* mutations are highly prevalent in clonal hematopoiesis of indeterminate potential [11], suggesting that *TET2* lesions are early driver events with the potential to predispose for further malignant transformation. *TET2* is involved in the epigenetic control of gene regulation, HSC self-renewal, and myeloid lineage commitment [12]. Deletion of *Tet2* in mice leads to sporadic development of MDS/CMML-like disease [13–16] and co-expression of mutated *Tet2* with additional oncogenic mutations, including *Asxl1, Ezh2, Jak2, c-Kit, Flt3, Bcor, Sf3b1*, and *AML1::ETO*, promotes disease in mice [12, 17, 18]. A recent work explored the co-occurrence of *Zrsr2* and *Tet2* mutations in the context of BPDCN [8]. They reported that the combined inactivation of *Zrsr2* and *Tet2* expands common myeloid progenitors (CMP), granulocyte-monocyte progenitors (GMP), and megakaryocyte-erythroid progenitors (MEP) compartments in in vivo transplantation experiments. To the best of our knowledge, no studies have explored the concurrence of *Zrsr2* and *Tet2* in MDS.

To investigate the functional role of *Zrsr2* and *Tet2* mutations in the context of hematopoiesis and MDS, we generated a novel mutant *Zrsr2* allele, either alone or in combination with *Tet2* deficiency. Here, we show that concurrent mutations in *Zrsr2* and *Tet2* promote MDS via dysregulation of gene expression and alternative splicing. We detect a disruption in myelo-erythroid differentiation and an expansion of LT-HSC and MPP2 compartments. Notably, 20–25% of double-mutant mice developed a more severe phenotype characterized by cytopenias (thrombocytopenia with or without anemia), splenomegaly with extramedullary hematopoiesis, and multi-lineage dysplasia, signs reminiscent of MDS. Moreover, we identify global gene expression changes and aberrant mRNA splicing of key biological pathways. Taken together, we demonstrate that mutations in *Zrsr2* and *Tet2* promote MDS with reduced penetrance.

## Materials and methods

### Animals

Novel *Zrsr2* mutant mice were created by CRISPR/Cas9 technology. In particular, exon 10 was targeted and a 17-nucleotide deletion was introduced, which generated a frameshift mutation [19]. Further details are provided in Supplementary information. Mice were then crossed to obtain homozygous mutants (*Zrsr2*^*m/m*^ and *Zrsr2*^*m/y*^) and wild-type (*Zrsr2*^*+/+*^ and *Zrsr2*^*+/y*^) animals. Both female and male *Zrsr2* mutant mice were used for experiments. To simplify nomenclature, we referred to all of them with the female genotypes. To generate animals carrying alterations in both *Zrsr2* and *Tet2, Zrsr2* mutant mice were crossed with a *Tet2* KO line previously described [20]. Both female and male double mutant animals were used for experiments. Mice were genotyped by standard PCR using *Zrsr2* and *Tet2* specific primers (Supplementary Table 1). CD45.1 mice, used as recipients in transplantation experiments, were purchased from Charles Rivers (Wilmington, MA, USA). Pilot preliminary experiments were performed for sample size estimates. No animals were excluded from the analysis. Regarding randomization, in all cases where multiple experimental groups were analyzed, mice were not randomly distributed but rather allocated in such a way that each group was evenly matched with regard to age range and frequency of mice of each sex. Investigators were not blinded to the mouse genotypes. This study was approved by the Regional Valencian Ministry of Agriculture (permit 2017/VSC/PEA/00200) and the Ethics Committee at IIS La Fe (permit 2016/0756).

### Flow cytometry analysis and cell sorting

Mice were sacrificed, autopsied, and bones (femurs, tibias, and cristae), spleen, and thymus were collected for further analysis. Red blood cells were lysed with Gey’s Solution (NH_4_Cl 8.3 g/L, NaHCO_3_ 1.0 g/L, EDTA 37 mg/L) and RBC-depleted cells were stained using monoclonal antibodies for 15 min at 4 °C. PB samples were lysed with BD FACS™ Lysing Solution (BD, Franklin Lakes, NJ, USA) and stained with monoclonal antibodies for 15 min at 4 °C. Lin^−^Sca-1^+^c-Kit^+^ (LSK) cells were sorted from a pool of 3 mice for whole transcriptome analysis and transplantation assays. BM cells were lysed with Gey’s solution, stained with anti-mouse CD117 MicroBeads (Miltenyi Biotec, Bergisch Gladbach, Germany), and enriched using LS columns (Miltenyi Biotec). c-Kit^+^ cells were subsequently stained with monoclonal antibodies for cell separation. Please refer to the Supplementary Information for additional details on antibodies and marker combinations.

### In vivo transplantation assays

For HSPC transplantations, LSK cells were FACS-sorted from a pool of 3 donor mice, and 8000 LSK cells (CD45.2) were transplanted along with 2 × 10^5^ support unfractionated bone marrow cells (CD45.1) into each lethally irradiated (12 Gray, split dose) mouse (CD45.1). Recipient mice were given enrofloxacin (Bayer, Leverkusen, Germany) in the drinking water for 4 weeks post-irradiation. PB engraftment was monitored monthly by flow cytometry.

### Gene expression analysis by RT-qPCR

Total RNA was extracted by RNeasy Mini Kit (QIAGEN, Hilden, Germany) from 10 × 10^6^ bone marrow or spleen cells previously lysed in RLT buffer (QIAGEN) supplemented with 1% β-mercaptoethanol (Sigma-Aldrich, St. Louis, MO, USA) and stored at −80 °C. RNA was treated with DNA-free DNase Treatment and Removal Reagents (Ambion, Austin, TX, USA). DNase-treated total RNA (1 µg) was reverse transcribed with oligo(dT) and TaqMan Reverse Transcription Reagents (Thermo Fisher Scientific, Waltham, MA, USA). The resulting cDNA was diluted tenfold before use. Real-time quantitative PCR was performed in technical triplicates using AceQ SYBR qPCR Master Mix (Vazyme Biotech Co., Nanjing, China) and the corresponding primers (Supplementary Table 1) using a ViiA 7 Real-Time PCR System (Thermo Fisher Scientific). All RT–qPCR analyses were performed on an Applied Biosystems QuantStudio Software V1.3 (Thermo Fisher Scientific). Primer set efficiency was firstly calculated in a standard curve experiment. Relative gene expression levels were calculated using the comparative C_T_ method. Since target and housekeeping primer set efficiencies differed >10%, we applied the corrective formula described in [21] to calculate the relative expression ratio. Gene expression levels were then normalized to that of *Hprt*.

### RNA sequencing

LSK cells (50,000 per sample) from pools of WT (*n* = 4), *Zrsr2*^*m/m*^ (*n* = 5), *Tet2*^*−/−*^ (n = 5), and *Zrsr2*^*m/m*^*Tet2*^*−/−*^ (*n* = 6) were sorted into RLT buffer supplemented with 1% β-mercaptoethanol. Total RNA extraction was performed using an RNEasy Micro kit (QIAGEN). DNA was then removed using on-column DNAse treatment. RNA integrity was assessed using the RNA Nano 6000 Assay Kit of the Bioanalyzer 2100 system (Agilent Technologies, CA, USA). Samples with an RNA Integrity Number value ≥7 were selected for further analysis. Low input directional RNA-seq libraries were prepared using NEBNext Ultra Directional RNA Library Prep Kit for Illumina (Illumina Inc., Cat N°. 7420, San Diego, CA, USA). Purified libraries were paired-end sequenced on the Illumina NovaSeq 6000 (Novogene, Beijing, China).

The quality of raw RNA-seq data was assessed in fastq data files using Fastp. Reads were mapped to the reference mouse genome GRCm38 (mm10), with genome annotations from GENCODE vM24, using the splicing aware mapping software STAR v2.5 [22]. Quantification of reads was calculated with HTSeq v0.6.1 [23]. Differential gene expression was determined using DESeq2 v2_1.6.3 [24] with adjusted *P* value ≤ 0.05, log2FoldChange ≥ 0. Differential alternative splicing was assessed using rMATS v3.2.1 [25]. This approach categorizes splicing events into five groups: retained intron (RI), skipped exon (SE), alternative 5′ splice site (A5SS), alternative 3′ splice site (A3SS), and mutually exclusive exons (MXE). Splicing events associated with each pair-wise comparison were identified based on false discovery rate (FDR) ≤ 0.05. Functional enrichment within lists of differentially spliced genes was identified with KEGG [26] and the enrichment cut-off *p* value was set as adjusted *P* value ≤ 0.05. RNA-seq data have been deposited in the ArrayExpress database at EMBL-EBI (www.ebi.ac.uk/arrayexpress) under accession number E-MTAB-10481.

The protein–protein interaction network between MAPK mis-spliced targets and related proteins was retrieved from STRING (http://string-db.org) [27]. The minimum required interaction score was set in high confidence (0.700). Network type was set in full STRING network (the edges indicate both functional and physical protein associations).

## Results

### Development of a germline *Zrsr2* mutant mouse

**Fig. 1:**
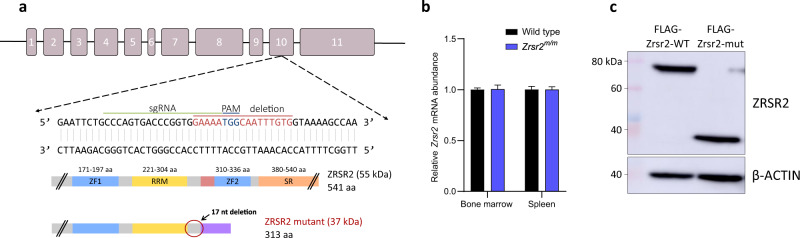
Generation of a germline *Zrsr2* allele. **a** Upper panel: *Zrsr2* gene consists of eleven exons (boxes). Middle panel: CRISPR/Cas9 construction targeting *Zrsr2* exon 10. Lines indicate gRNA sequence (green), PAM motif (blue), and the resulting 17-nucleotides deletion (red). Lower panel: Diagram of WT and truncated ZRSR2 protein with their functional domains: ZF (Zinc Finger), RRM (RNA recognition motif), SR (Serine/Arginine rich domain). Mutant *Zrsr2*^*m/m*^ mice expressed a mutated short ZRSR2 protein containing a ZF domain and the RRM domain. The numbers indicate amino acids encoded by WT and mutant protein as predicted in silico. **b** Relative mRNA abundance of *Zrsr2* in bone marrow and spleen determined by RT-qPCR. Data normalized to *Hprt*. BM: WT (*n* = 9), *Zrsr2*^*m/m*^ (*n* = 10); SPL: WT (*n* = 6), *Zrsr2*^*m/m*^ (*n* = 6). **c** Western blot of FLAG-tagged WT and truncated ZRSR2 protein overexpression in HEK293T cells. Proteins were detected using an anti-FLAG antibody in total cell lysates. Data from two independent experiments.

To gain insight into the role of *Zrsr2* in normal and malignant hematopoiesis, we generated a mutant allele targeting *Zrsr2* in vivo *by* CRISPR/Cas9 technology (Fig. 1a). This approach generated a 17-nucleotide deletion in exon 10 of *Zrsr2* DNA sequence (Supplementary Fig. 1a), which resulted in a frameshift mutation. Consequently, the mutated *Zrsr2* allele lacks the second zinc finger domain and the serine-rich domain (Fig. 1a). Seventeen nucleotide deletion was confirmed in different hematopoietic tissues (Supplementary Fig. 1b). Mutant bone marrow and spleen cells expressed levels of *Zrsr2* mRNA equivalent to WT controls, suggesting that the mutated allele escapes the nonsense-mediated mRNA decay (Fig. 1b). In addition, 17-nucleotide deletion, in turn, caused skipping of exon 10, spliced event that was confirmed by RT-PCR in cDNA from LSK cells (Supplementary Fig. 1c). Due to difficulties to detect ZRSR2 protein in murine tissue, we tagged the WT and truncated mutant protein with FLAG and over-expressed it in HEK293T cells. Both WT and mutant ZRSR2 forms were detected, indicating that the truncated protein product is stable (Fig. 1c). Hemizygous *Zrsr2*^*m/y*^ or homozygous *Zrsr2*^*m/m*^ mice were mated with *Tet2* knockout mice to produce *Zrsr2*^*m/m*^*Tet2*^*−/−*^ double mutant mice. *Zrsr2* mutant mice had a normal lifespan (24 months) while double mutant mice died at earlier ages (average 14 months). *Zrsr2*^*m/m*^ females were infertile.

### *Zrsr2* mutation and *Tet2* loss expand LT-HSC and MPP2 compartments and impair myelo-erythroid progenitor differentiation in steady-state

**Fig. 2:**
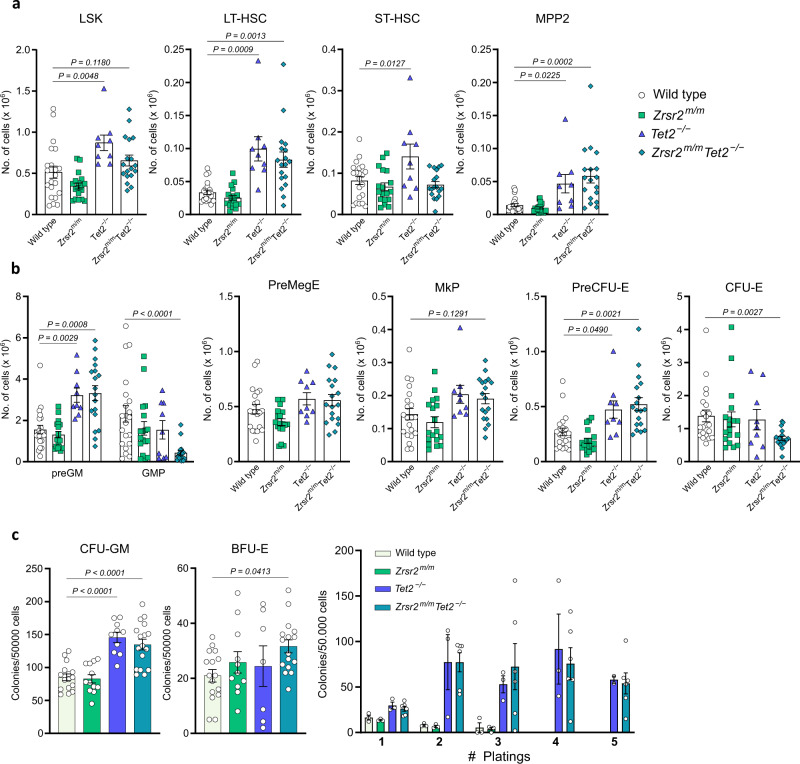
*Zrsr2* mutation and *Tet2* loss expand LT-HSC and impair myelo-erythroid differentiation. **a** Flow cytometry analysis of the LSK compartment from 3-months-old WT (*n* = 21), *Zrsr2*^*m/m*^ (*n* = 18), *Tet2*^*−/−*^ (*n* = 9), and *Zrsr2*^*m/m*^*Tet2*^*−/−*^ mice (*n* = 18). Representative dot plots are shown in Supplementary Fig. 2. **b** Flow cytometry analysis of myelo-erythroid progenitors from 3-months-old WT (*n* = 21), *Zrsr2*^*m/m*^ (*n* = 18), *Tet2*^*−/−*^ (*n* = 9), and *Zrsr2*^*m/m*^*Tet2*^*−/−*^ (*n* = 18) mice. Representative dot plots are shown in Supplementary Fig. 2. **c** Left and middle panel: GM-CFU and BFU-E forming capacity of unfractionated BM from 3-month-old WT (*n* = 18), *Zrsr2*^*m/m*^ (*n* = 12), *Tet2*^*−/−*^ (*n* = 10), and *Zrsr2*^*m/m*^ *Tet2*^*−/−*^ (*n* = 17) mice. Right panel: Serial replating capacity of unfractionated BM from 3-months-old WT (*n* = 3), *Zrsr2*^*m/m*^ (*n* = 3), *Tet2*^*−/−*^ (*n* = 3), and *Zrsr2*^*m/m*^ *Tet2*^*−/−*^ (*n* = 6) mice cultured under myeloid conditions. Data represent mean ± SEM.

We analyzed the stem and myelo-erythroid progenitor compartments at 12-13 weeks. Fractionation of the HSC (LSK) compartment using CD150 and CD48 expression showed expansion of some phenotypic HSC subsets, such as the long-term HSC (LT-HSC, LSK CD150^+^CD48^*−*^) [28] and the multipotent MPP2 progenitors (LSK CD150^+^CD48^+^, erythroid, and megakaryocyte biased progenitors) [29, 30] (Fig. 2a, Supplementary Fig. 2a). At the progenitor level, we observed a significant increase in the early precursors of granulo-monocytic (PreGM) and erythroid (PreCFU-E) progenitors, an increase in megakaryocytic progenitors (MkP) and a decrease in the more committed granulo-monocytic (GMP), and erythroid (CFU-E) progenitors, suggestive of a differentiation block at the early stages of myelo-erythroid lineage commitment [31] (Fig. 2b, Supplementary Fig. 2b). These phenotypic alterations were accompanied by an increase in GM-CFU and BFU-E colony-forming units in *Zrsr2*^*m/m*^*Tet2*^*−/−*^ mice (Fig. 2c). *Zrsr2*^*m/m*^*Tet2*^*−/−*^ myeloid progenitors exhibited properties of self-renewal as evidenced by sustained CFU activity in serial replating assays (Fig. 2c). Overall, we observed a general skew towards myeloid lineage commitment at expense of the B-lymphoid lineage (Supplementary Fig. 2c). No effects on T lymphopoiesis were observed in single or double mutant mice (Supplementary Fig. 2d).

### *Zrsr2* mutation and *Tet2* loss result in MDS

**Fig. 3:**
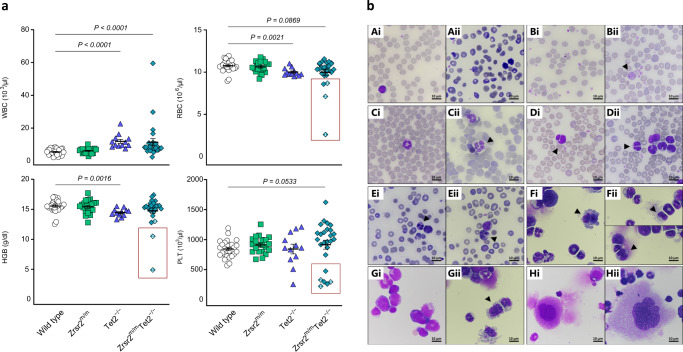
Concomitant *Zrsr2* mutation and *Tet2* loss result in PB cytopenias and multilineage dysplasia. **a** Hematological parameters from 3-month-old WT (*n* = 28), *Zrsr2*^*m/m*^ (*n* = 21), *Tet2*^*−/−*^ (*n* = 12), and *Zrsr2*^*m/m*^*Tet2*^*−/−*^ mice (*n* = 26). WBC: white blood cells, RBC: red blood cells, HGB: hemoglobin, PLT: platelets. Boxes show mice presenting cytopenias. **b** Peripheral blood smears and bone marrow cytospins from 3-months-old WT and *Zrsr2*^*m/m*^*Tet2*^*−/−*^ mice stained with May-Grünwald Giemsa. Normal RBC from WT (Ai) and dysplastic RBC from *Zrsr2*^*m/m*^*Tet2*^−/−^ mice (Aii) showing RBC anisocytosis, polychromasia, and presence of nucleated erythrocytes. Normal platelets from WT (Bi) and dysplastic platelets from *Zrsr2*^*m/m*^*Tet2*^*−/−*^ mice (Bii) showing anisocytosis and giant size. Normal neutrophil from WT (Ci), hypersegmented neutrophil (Cii) and hyposegmented neutrophils with pseudo Pelger-Huët anomalies (Di–Dii) from *Zrsr2*^*m/m*^*Tet2*^−/−^ mice. Nuclear fragmentation and karyorrhexis of granulocytes (Ei-Eii). Erythrophagocytosis (Fi) and binucleated erythroid precursors (Fii) in BM of *Zrsr2*^*m/m*^*Tet2*^−/−^ mice. Normal myeloid precursors from WT (Gi) and dysplastic myeloid precursors with cytoplasm vacuolization from *Zrsr2*^*m/m*^*Tet2*^−/−^ mice (Gii). Normal megakaryocyte in WT (Hi) and giant megakaryocyte in *Zrsr2*^*m/m*^*Tet2*^−/−^ mice (Hii). Dysplastic features are indicated with arrows.

**Fig. 4:**
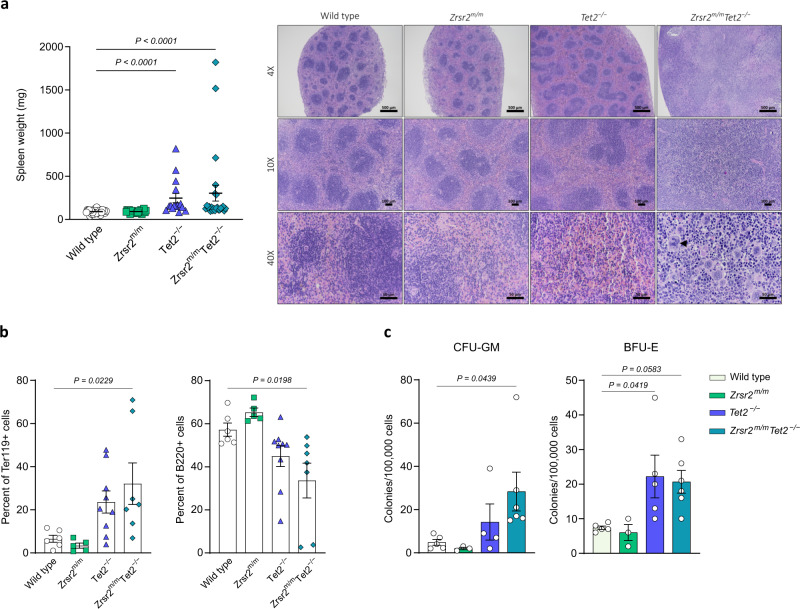
Concomitant *Zrsr2* mutation and *Tet2* loss result in splenomegaly with extramedullary hematopoiesis. **a** Left panel: Spleen weight from 3-months-old WT (*n* = 25), *Zrsr2*^*m/m*^ (*n* = 21), *Tet2*^*−/−*^ (*n* = 9), and *Zrsr2*^*m/m*^*Tet2*^−/−^ (*n* = 24) mice. Right panel: Spleen sections from 3-months-old WT (*n* = 3), *Zrsr2*^*m/m*^ (*n* = 2), *Tet2*^−/−^ (*n* = 3), and *Zrsr2*^*m/m*^*Tet2*^−/−^ (*n* = 6) mice stained with H&E. The arrow indicates presence of megakaryocytes with dysplastic changes. **b** Percentage of erythroid (Ter119^+^) and B cells (B220^+^) in spleen of 3-months-old WT (*n* = 6), *Zrsr2*^*m/m*^ (*n* = 5), *Tet2*^−/−^ (*n* = 9), and *Zrsr2*^*m/m*^*Tet2*^−/−^ (*n* = 7) mice. **c** GM-CFU and BFU-E forming capacity of spleen cells from 3-month-old WT (*n* = 5), *Zrsr2*^*m/m*^ (*n* = 3), *Tet2*^−/−^ (*n* = 5), and *Zrsr2*^*m/m*^*Tet2*^−/−^ (*n* = 6) mice. Data represent mean ± SEM.

Notably, within the cohort of *Zrsr2*^*m/m*^*Tet2*^*−/−*^ mice analyzed, a subset of mice (25%, 6 out of 24) showed a more aggressive hematological phenotype. These mice presented cytopenias (thrombocytopenia with or without anemia) (Fig. 3a). Evaluation of peripheral blood smears and bone marrow cytospins indicated that *Zrsr2*^*m/m*^*Tet2*^*−/−*^ mice presented multi-lineage dysplastic features (Fig. 3b). We observed signs of dyserythropoiesis including RBC anisocytosis, polychromasia, and the presence of circulating nucleated red cells (Fig. 3b/Aii). In addition, we observed erythrophagocytosis and binucleated erythroid precursors in the bone marrow (Fig. 3b/Fi, Fii). Megakaryocytic dysplasia included platelet anisocytosis, the presence of abnormally large platelets in peripheral blood (Fig. 3B/Bii), and giant megakaryocytes in the bone marrow (Fig. 3b/Hii). Myeloid dysplasia was present in the form of neutrophil hypersegmentation (Fig. 3b/Cii), granulocyte hyposegmentation with pseudo Pelger-Huët anomalies (Fig. 3b/Di, Dii), nuclear fragmentation and karyorrhexis of neutrophils, and abnormal vacuolization in myeloid precursors (Fig. 3b/Gii). Macroscopic analysis of *Zrsr2*^*m/m*^*Tet2*^*−/−*^ spleens showed splenomegaly (Fig. 4a). Histopathological analysis of splenic sections demonstrated disruption of red and white pulp in *Zrsr2*^*m/m*^*Tet2*^*−/−*^ mice (Fig. 4a). A closer examination revealed infiltration by megakaryocyte and erythroid (Ter119^+^) cells and a decrease in B cells (B220^+^) as assessed by flow cytometry (Fig. 4b). Signs of extramedullary hematopoiesis were evidenced by the presence of GM-CFU and BFU-E activity in the spleen of *Zrsr2*^*m/m*^*Tet2*^*−/−*^ mice (Fig. 4c). Overall, a subset of *Zrsr2*^*m/m*^*Tet2*^*−/−*^ mice showed features consistent with myelodysplasia [32].

Since MDS is a disease of the elderly, we also analyzed the impact of mutations in aged control (24 months), *Zrsr2*^*m/m*^ (24 months), and *Zrsr2*^*m/m*^*Tet2*^*−/−*^ mice (18 months). Old *Zrsr2*^*m/m*^ mice presented anemia and one out three cases had leukocytosis, thrombocytopenia, and splenomegaly with disrupted spleen histology. *Zrsr2*^*m/m*^*Tet2*^*−/−*^ mice displayed anemia and thrombocytopenia (Supplementary Fig. 3a), and exhibited erythroid and myeloid dysplasia (Supplementary Fig. 3b). This was accompanied by splenomegaly with disrupted splenic architecture (Supplementary Fig. 3c, d). Notably, aged *Zrsr2*^*m/m*^*Tet2*^*−/−*^ mice were analyzed at earlier time-points (18 months) due to evident signs of illness (reduced mobility, hunched posture, ruffled fur, weight loss, and white foot paws). Interestingly, 1 out of 6 aged *Zrsr2*^*m/m*^*Tet2*^*−/−*^ mice showed signs suggestive of progression to secondary AML, such as an increase of blasts in PB smears and myeloperoxidase (MPO) staining in spleen and liver sections (Supplementary Fig. 3e–g).

### Zrsr2 mutation and *Tet2* loss in HSPC enhance BM repopulation capacity and cause cell-autonomous hematopoietic alterations

**Fig. 5:**
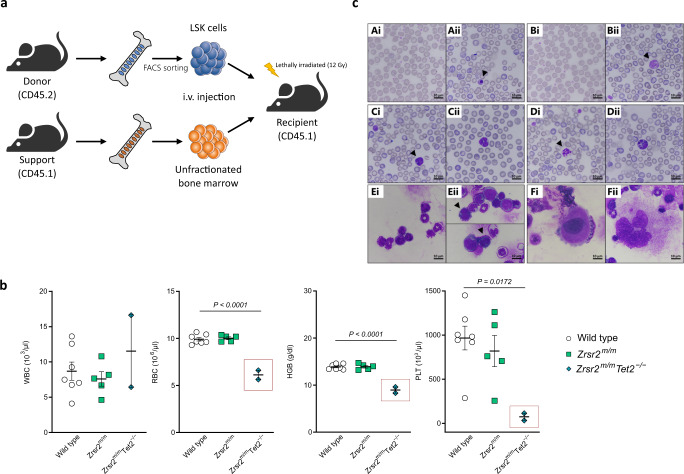
Combined *Zrsr2-Tet2* mutations in HSPC cause cell-autonomous hematopoietic alterations. **a** Experimental design for transplantation. Lethally irradiated CD45.1 recipient mice were co-transplanted with 8.000 LSK (CD45.2, isolated from each genotype) and 200.000 support unfractionated bone marrow (CD45.1) cells. Peripheral blood was analyzed monthly by flow cytometry after transplantation. **b** Hematological parameters in transplanted animals at 15 months post-transplantation. WT (*n* = 7), *Zrsr2*^*m/m*^ (*n* = 5), and *Zrsr2*^*m/m*^*Tet2*^*−/−*^ (*n* = 2). Boxes show mice presenting cytopenias. **c** May-Grünwald Giemsa staining of peripheral blood smears and bone marrow cytospins in transplanted animals at 15 months post-transplantation. Normal RBC from WT (Ai) and dysplastic RBC from *Zrsr2*^*m/m*^*Tet2*^*−/−*^ (Aii), showing RBC anisocytosis, polychromasia, and a nucleated RBC. Normal platelets from WT (Bi) and giant platelet from *Zrsr2*^m/m^*Tet2*^−/−^ mice (Bii). Hyposegmented granulocytes (Ci-Cii) and granulocytes with nuclear fragmentation and karyorrhexis (Di–Dii) from *Zrsr2*^m/m^*Tet2*^−/−^ mice. Normal progenitors from WT (Ei) and binucleated erythroid precursors from *Zrsr2*^m/m^*Tet2*^−/−^ mice (Eii). Megakaryocyte from WT (Fi) and enlarged megakaryocyte from *Zrsr2*^m/m^*Tet2*^−/−^ mice (Fii). (A-F) Data represent mean ± SEM and come from three independent experiments.

Since there is evidence of HSPC involvement in MDS, we evaluated hematopoietic stem cell function in *Zrsr2* mutant and *Zrsr2*^*m/m*^*Tet2*^*−/−*^ mice. We performed transplantation assays using 8000 LSK cells (CD45.2) along with 2 × 10^5^ support unfractionated bone marrow cells (CD45.1) (Fig. 5a). 8–12 weeks after transplantation, the reconstitution of the recipient (CD45.1) hematopoietic system by the donor cells was verified by flow cytometric analysis of the peripheral blood. We observed that *Zrsr2*^*m/m*^*Tet2*^*−/−*^ cells displayed an increased repopulation capacity and retained multi-lineage reconstitution ability (Supplementary Fig. 4a). In the donor-derived CD45.2^+^ fraction, we detected an increase in erythroid-megakaryocytic biased MPP2 progenitors and a decrease in PreMegE, suggestive of a differentiation block along the erythroid lineage at an earlier step (Supplementary Fig. 4b, c). As occurred in steady-state, we also observed an accumulation of PreGM progenitors and a decrease in GMP (Supplementary Fig. 4c). LT-HSC were reduced, suggesting a push of LT-HSC towards more committed HSPC in a transplantation context. Further, *Zrsr2*^*m/m*^*Tet2*^*−/−*^ mice presented cytopenias (anemia and thrombocytopenia) (Fig. 5b) and myeloid and erythroid dysplasia (Fig. 5c). Remarkably, transplanted *Zrsr2*^*m/m*^*Tet2*^*−/−*^ animals were moribund at the time of sacrifice (15 months post-transplantation).

### Whole transcriptome sequencing in HSPC shows an alteration of lineage-specific hematopoietic genes and an enrichment of inflammatory pathways

**Fig. 6:**
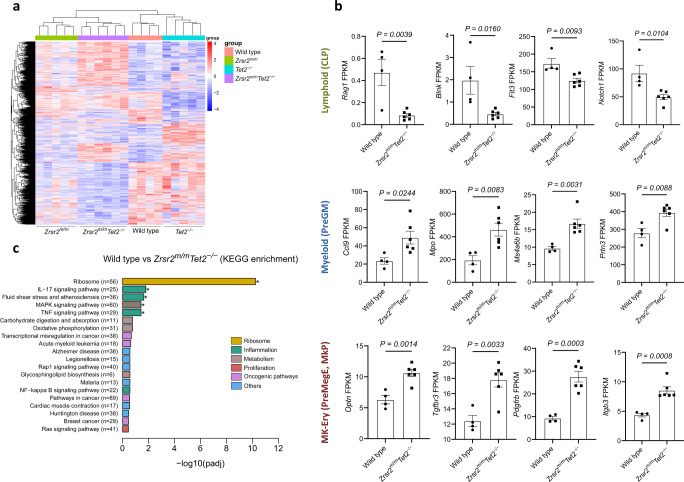
Gene expression dysregulation in *Zrsr2*^*m/m*^*Tet2*^*−/−*^ LSK cells leads to transcriptional myeloid priming and enrichment of inflammatory pathways. **a** Heatmap visualizing the differentially expressed genes (DEG) identified in WT, *Zrsr2*^*m/m*^, *Tet2*^*−/−*^, and *Zrsr2*^*m/m*^*Tet2*^*−/−*^ LSK cells from 3-months-old mice (red, up-regulated genes; blue, down-regulated genes). *n* = 4-6 replicates/genotype. **b** Gene expression levels of CLP, PreGM, and PreMegE and MkP associated genes. **c** KEGG enrichment analysis of DEG in LSK from WT vs *Zrsr2*^*m/m*^*Tet2*^*−/−*^ mice.

Next, we performed RNA sequencing experiments to get insight into the molecular changes of *Zrsr2*^*m/m*^*Tet2*^*−/−*^ HSPC. Transcriptome analysis resulted in distinct gene expression signatures (Fig. 6a). Differential gene expression analysis in pair-wise comparisons against WT control by DESEq2 [24] yielded 571 differentially expressed genes (DEG) in *Zrsr2* mutant, 1203 DEG in *Tet2*^−/−^ and 2952 DEG in *Zrsr2*^*m/m*^*Tet2*^*−/−*^ LSK cells (adjusted *P*-value ≤ 0.05). To evaluate the impact of the combined dysfunction of *Zrsr2* and *Tet2* on DNA methylation, we performed global epigenomic profiling of WT, *Tet2*^*−/−*^, and *Zrsr2*^*m/m*^*Tet2*^*−/−*^ mice using Infinium Mouse Methylation Bead Chip array (285K, Illumina). Principal component analyses (PCA) revealed that *Zrsr2*^*m/m*^*Tet2*^*−/−*^ and *Tet2*^−/−^ hematopoietic cells had distinct epigenomic profiles from those of WT (Supplementary Fig. 5a). We observed that 8747 CpG, corresponding to 531 murine genes, were found differentially methylated in *Zrsr2*^*m/m*^*Tet2*^*−/−*^ cells in comparison to WT cells (Supplementary Table 2). Next, we performed a Venn diagram analysis to identify the overlapping genes in both transcriptomic and epigenomic analyses. We detected that circa 3% of DEG presented alterations in DNA methylation levels (Supplementary Fig. 5b, Supplementary Table 4a). Within *Zrsr2*^*m/m*^*Tet2*^*−/−*^ LSK, 1327 genes were up-regulated and 1625 genes were down-regulated. Molecularly, *Zrsr2*^*m/m*^*Tet2*^*−/−*^ LSK were primed towards a myeloid fate since transcripts related to common lymphoid progenitors (*Rag1, Blnk, Flt3*) and the Notch1 pathway were down-regulated (Fig. 6b) while transcripts associated to preGM (*Ccl9, Mpo, Ms4a6b, Prtn3*) (Fig. 6b), preMegE (*Optn, Tgfbr3*), and MkP (*Pdgfrb, Itgb3*) [33] were up-regulated (Fig. 6b). Interestingly, gene expression changes in *Blnk, Mpo, Tgfbr3* are potentially deregulated by DNA methylation changes (Supplementary Table 4b). This suggests that myeloid and megakaryocyte-erythroid priming originates in the immature HSPC in concordance with the increased proportion of erythroid-megakaryocytic biased progenitors (MPP2) within the LSK compartment. Enrichment analysis of DEG using GO indicated that genes up-regulated in *Zrsr2*^*m/m*^*Tet2*^*−/−*^ LSK were associated to ribosome, inflammation, and cell migration/motility processes (Supplementary Fig. 5c). Kyoto Encyclopedia of Genes and Genomes (KEGG) pathway enrichment analysis [26] in *Zrsr2*^*m/m*^*Tet2*^*−/−*^ LSK showed up enrichment in ribosome function and pro-inflammatory cytokine pathways, such as those mediated by IL-17, TNF-α, and mitogen-activated protein kinase (MAPK) signaling (Fig. 6c). MDS is frequently associated with deregulated inflammatory signaling profiles in BM [34]. On this basis, we conducted a cytokine multiplex assay and found that mice displaying a more aggressive MDS phenotype presented higher pro-inflammatory cytokines levels (IFN-γ, IL-6, IL-10, and TNF-α), suggesting a contributing role of inflammation in *Zrsr2*^*m/m*^*Tet2*^*−/−*^ MDS phenotype (Supplementary Fig. 6).

### Aberrant mRNA splicing in *Zrsr2*^*m/m*^*Tet2*^*−/−*^ mice target the MAPK protein family

**Fig. 7:**
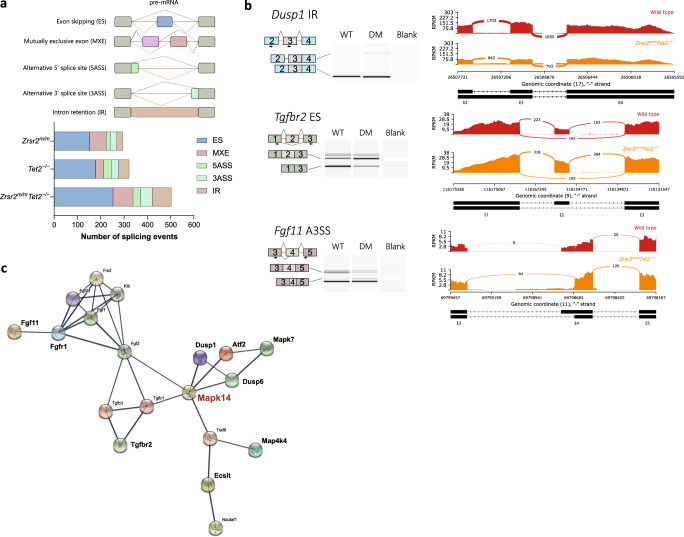
Aberrant alternative splicing in *Zrsr2*^*m/m*^*Tet2*^*−/−*^ LSK cells targets the MAPK pathway. **a** Number of significant aberrant splicing events in *Zrsr2*^*m/m*^, *Tet2*^−/−^, and *Zrsr2*^*m/m*^ *Tet2*^*−/−*^ LSK cells (*P* value ≤ 0.01, FDR ≤ 0.05). **b** Validation of relevant aberrant splicing events identified by RNA-seq using RT- PCR. Sashimi plots showing an IR event in *Dusp1*, an ES event in *Tgfbr2*, and an A3SS event in *Fgf11*. **c** Protein-protein interactions among MAPK-related targets were retrieved from STRING. Line thickness represents interaction confidence and edges indicate both functional and physical protein associations. Relevant mis-spliced targets identified in this study (in bold) converge in the MAPK14 (red).

To determine alternative splicing patterns in WT, *Zrsr2*^*m/m*^, *Tet2*^−/−^, and *Zrsr2*^*m/m*^*Tet2*^*−/−*^ LSK cells we employed rMATS [25]. Alternative spliced events were categorized into five major types: skipped exon (SE), MXE, A5SS, A3SS, and RI. Using pair‐wise comparisons against WT control, we identified 294 significant differentially spliced events in *Zrsr2*^*m/m*^, 322 in *Tet2*^−/−^, and 503 in *Zrsr2*^*m/m*^*Tet2*^*−/−*^ (*P* value ≤ 0.01, FDR ≤ 0.05) (Fig. 7a, Supplementary Table 3). We found that 253 alternative spliced genes were uniquely present in *Zrsr2*^*m/m*^*Tet2*^*−/−*^ cells, implying that the combined alteration of *Zrsr2* and *Tet2* cooperatively promoted mis-splicing of these transcripts (Supplementary Fig. 7a). KEGG pathway enrichment analysis was performed on differentially spliced genes (Supplementary Fig. 7b). We found that the MAPK kinase family and the Fanconi anemia pathway were the most affected targets by mRNA aberrant splicing in *Zrsr2*^*m/m*^*Tet2*^*−/−*^. Importantly, a total of nine genes related to the MAPK pathway were identified as mis-spliced, from which we validated 3, *Dusp1*, *Tgfbr2*, and *Fgf11* (Fig. 7b). Strikingly, we observed that these targets converged into a particular kinase, the MAPK14 (Fig. 7c). We also identified and corroborated exon skipping events in *Per1* and *Ttbk2* (in *Zrsr2*^*m/m*^*Tet2*^*−/−*^), and *Frrs1* (in *Zrsr2*^*m/m*^) transcripts, involved in circadian regulation, cell migration/motility, and iron metabolism, respectively (Supplementary Fig. 7c). Remarkably, mis-splicing of these targets was also found in human *ZRSR2* and *ZRSR2-TET2* co-mutated samples (unpublished observations).

## Discussion

Here we show that mutations of *Zrsr2* and *Tet2*, genes commonly co-mutated in human MDS, resulted in impairment of myelo-erythroid differentiation and the development of thrombocytopenia and anemia, multi-lineage dysplasia, and splenomegaly with extramedullary hematopoiesis. These features, manifested with incomplete penetrance, are compatible with MDS [32]. Further, *Zrsr2* and *Tet2* mutations were associated with an expansion of LT-HSC and MPP2 compartments and an increase of CFUs, with CFU-GM cells acquiring self-renewal abilities. Moreover, the phenotype observed in steady-state mice was reproduced in transplanted mice, thus indicating that these alterations were cell-autonomous. Altogether, *Zrsr2* and *Tet2* mutations cause a phenotype that recapitulates critical features of myelodysplasia.

Prior studies exploring in vivo *Zrsr2* function in hematopoiesis provided somewhat contrasting results. *Zrsr2* constitutive knockout mice exhibited normal myeloid development and regular repopulating ability [6]. Data from mice with hematopoietic-specific conditional deletion of *Zrsr2* showed that *Zrsr2* loss promotes HSC self-renewal [7]. In our study, young adult mice expressing truncated ZRSR2 (*Zrsr2*^*m/m*^) exhibited normal myeloid development and regular repopulating ability, similar to the phenotype described in *Zrsr2* constitutive knockout [6]. However, old *Zrsr2*^*m/m*^ mice did display several hematological alterations, such as reduced RBC and hemoglobin levels akin to patients with isolated *ZRSR2* mutations showing refractory macrocytic anemias [35]. One aspect for which our model differs from previous studies is that we did not generate a knockout but a stable truncated protein. Many *Zrsr2* mutations observed in the clinics are likely analogous to the *Zrsr2* mutant allele that we describe here in that they generate a loss of the C-terminal region [3]. Other models of MDS based on mutations in recurrently mutated genes have been described. *Sf3b1*^*+/K700E*^*Tet2*^*−/−*^ mice [18] showed similar characteristics to the ones we report here, such as anemia, expansion of the LT-HSC compartment, and erythroid and megakaryocyte dysplasia. Other studies recapturing MDS/MPN-like disease also showed splenomegaly with extramedullary hematopoiesis [36]. Loss of *Tet2* itself promotes myeloid-biased hematopoiesis and HSC self-renewal [15]. Correspondingly, we show that concurrent deletion of *Tet2* enhanced myeloid-biased differentiation, as reflected by increased MPP2 progenitors and increased myelo-erythroid lineage priming in LSK HSPC. Further, *Tet2* loss confers growth advantage and self-renewal capacity to *Zrsr2* mutant progenitors, as evidenced by clonogenic and serial replating assays. This increased output of colonies has also been shown in an *Asxl1*^*−/−*^*Tet2*^*−/−*^ model [37]. In the transplantation setting, *Zrsr2*^*m/m*^*Tet2*^*−/−*^ cells showed higher repopulation capacity than *Zrsr2* mutant and WT cells, in accordance with the clonal advantage typical of MDS HSC. In addition, we observed an increased reconstitution to megakaryocytic-erythroid biased MPP2 progenitors, LSK, and PreGM in mice transplanted with *Zrsr2*^*m/m*^*Tet2*^*−/−*^ cells. Similarly, previous work in bone marrow chimeras transplanted with *Zrsr2* and *Tet2* double knockout cells reported an increase in LSK and myeloid progenitors (CMP, GMP, and MEP) [8].

Deregulated DNA methylation is one of the hallmarks of MDS [38], although in our study only circa 3% of genes that were differentially expressed in *Zrsr2*^*m/m*^*Tet2*^*−/−*^ LSK may potentially be regulated by an epigenetic mechanism such as DNA methylation. Importantly, transcriptional profiling followed by GO enrichment analysis indicated that genes upregulated in *Zrsr2*^*m/m*^*Tet2*^*−/−*^ LSK were associated with ribosome, inflammation, and cell migration/motility. Interestingly, alterations in ribosome biogenesis are associated with MDS and defective erythropoiesis [39, 40]. On the other hand, deregulation of inflammatory signaling plays a central role in the pathogenesis of MDS [34]. In fact, MDS patients present higher levels of pro-inflammatory cytokines in serum [41]. In this regard, splicing factor mutations and *Tet2* deficiency have been associated with increased inflammatory cytokine production [42, 43]. Cytokine multiplex assay reveals that *Zrsr2*^*m/m*^*Tet2*^*−/−*^ mice with more aggressive MDS phenotype display higher levels of pro-inflammatory cytokines. This suggests a possible role of inflammation favoring MDS.

Since mutations in *ZRSR2* alter RNA splicing and HSC are the cell-of-origin of MDS, we performed an alternative splicing analysis in LSK cells. Unexpectedly, this analysis showed that exon skipping but not intron retention was the category most affected in *Zrsr2* mutant mice. This contrasts with most of the literature associating *ZRSR2* to intron retention of U12-type introns [4–8]. However, our data are in agreement with one study reporting that *ZRSR2* mutations in humans cause more exon skipping than intron retention [44]. One of the most affected pathways by mis-splicing in *Zrsr2*^*m/m*^*Tet2*^*−/−*^ LSK cells was MAPK signaling. Among the nine genes that were found to be aberrantly spliced in this pathway, we confirmed *Dusp1*, *Tgfbr2*, and *Fgf11*. Interestingly, we found that these MAPK-related targets converged into MAPK14, an essential kinase for definitive erythropoiesis in mice [45]. In addition, MAPK14 has a key role in regulating pro-inflammatory cytokine production via MK2 and MSK1/2. Aberrant mRNA splicing in this pathway, thus, may deregulate MAPK14 function and explain, in combination with other transcriptional changes, the level of pro-inflammatory cytokines found in *Zrsr2*^*m/m*^*Tet2*^*−/−*^ mice. This finding coincides with previous work showing MAPK14 as a down-regulated target downstream of *ZRSR2* mutation [6].

Collectively, these data provide a unique insight into the mechanisms by which *Zrsr2* and *Tet2* mutations affect MDS pathogenesis. Our results indicate that concomitant mutations in *Zrsr2* and *Tet2* can initiate MDS in mice. We identify alterations in HSC function and myelo-erythroid differentiation as well as transcriptional and splicing changes in relevant biological pathways (MAPK family, Fanconi Anemia pathway) that may contribute to MDS tumorigenesis. Given these findings, MAPK modulators, anti-inflammatory agents, and drugs aiming to restore normal myelo-erythroid differentiation may be tested in our model. This is particularly important given the unmet clinical need to find new curative approaches beyond HSPC transplantation. Since co-occurrence of *ZRSR2* and *TET2* mutations have also been observed in BPDCN, our model could also be exploited to explore the biology of dendritic leukemia. Overall, this study expands on mouse models based on recurrent mutations in splicing and epigenetic regulators and offers an opportunity to elucidate further the mechanisms governing tumorigenesis in minor spliceosome-mutated MDS.

## Supplementary information

Supplemental Information

Supplementary table 2

Supplementary table 3
