# Supplementary material for: Concurrent Zrsr2 mutation and Tet2 loss promote myelodysplastic neoplasm in mice
Source: Leukemia. 2022 Aug 27;36(10):2509–18. doi: 10.1038/s41375-022-01674-2 (PMC9522584; doi:10.1038/s41375-022-01674-2)
Supplement: Supplementary file 1 — Supplemental Information [file 41375_2022_1674_MOESM1_ESM.docx]

**Supplementary information**

- **Supplementary methods**
- **Supplementary table 1.** Primers used in this study.
- **Supplementary table 2.** Related to DNA methylation studies (xlsx).
- **Supplementary table 3.** Related to alternative splicing analysis (xlsx).
- **Supplementary table 4.** Related to DNA methylation and transcriptomics data.
- **Supplementary Figure 1.** Related to generation of a germline *Zrsr2* allele.
- **Supplementary Figure 2.** FACS analysis of HSC, myelo-erythroid and B progenitors, and T lymphocytes.
- **Supplementary Figure 3.** Hematological phenotype of aged control (24 months), *Zrsr2* mutant (24 months) and *Zrsr2^m/m^Tet2^−/−^* mice (18 months).
- **Supplementary Figure 4.** Related to bone marrow transplantation of *Zrsr2-Tet2* HSPC.
- **Supplementary Figure 5.** Related to transcriptome and epigenetic analysis.
- **Supplementary Figure 6.** Levels of pro-inflammatory cytokines.
- **Supplementary Figure 7.** Analysis of aberrant splicing events identified by RNA-seq.
- **Supplementary references**

**Supplementary methods**

## Additional information regarding the generation of the *Zrsr2* mutant mice

Single guide RNA sequences (sgRNA) were designed using the Breaking Cas online software. To target the *Zrsr2 mouse gene (ENSMUSG00000031370)*, single guide RNA (sgRNA) (CCCAGTGACCCGGTGGAAAA) was designed against the exon 10 and was produced by *in vitro* transcription (MEGAshortscript T7 kit, Life Technologies) from g-Blocks containing T7 promoter. Capped polyadenylated Cas9 mRNA was produced by *in vitro* transcription (mMESSAGE mMACHINE T7 ULTRA kit, Life Technologies) from the plasmid pMJ920 (Addgene 42234) linearized with BstBI and treated with Antarctic phosphatase (NEB). Both the Cas9 mRNA and the sgRNA were purified using MEGAclear kit (Life Technologies) and eluted in TE buffer. Cas9 mRNA (50 ng/µl) and sgRNA (20 ng/ µl) were injected into one-cell embryos [1] obtained from the cross of female C57BL6J/CBAF1 with male C57BL6J. Microinjected embryos were allowed to develop to blastocysts stage and transferred into the uterus of CD1 pseudopregnant females to produce founder mice (F0) [2]. For genotyping, DNA was extracted from the tail of 3-week-old mice using a conventional DNA isopropanol-ethanol precipitation protocol. PCR products were visualized by 3% agarose gel electrophoresis. PCR products were sequenced by Sanger using DNA extracted from a tail biopsy using specific primers (sequences in **Supplementary Table S1**). Wild-type (*Zrsr2^+/y^ or Zrsr2^+/+^*) and homozygous mutant mice (*Zrsr2^m/y^ or Zrsr2^m/m^*) were analyzed after backcrossing to C57BL/6J background for six generations.

**Flow cytometry analysis**

For immunophenotyping, the following antibodies were used: CD4-PE (1:200, clone GK1.5, Miltenyi Biotec, #130-121-131), Ly-6G/Ly-6C/Gr-1-PE-Cy5 (1:800, clone RB6-8C5, Biolegend, #108409), CD8a-FITC (1:400, clone 53-6.7, Miltenyi Biotec, #[130-118-468](about:blank#copy-to-clipboard)), B220-PE-Cy7 (1:400, clone RA3-6B2, BD, #561881), CD11b/Mac-1-V450 (1:400, clone M1/70, BD, #560456), for mature cells; CD45.1-APC (1:200, clone A20, Biolegend, #110713), CD45.2-APC-Cy7 (1:200, clone 104, BD, #560694), for chimerism in PB; CD45.1-PE-Cy7 (1:200, clone A20, Biolegend, #110729), CD45.2-BV510 (1:25, clone 104, Biolegend, #109837), for chimerism in BM; lineage cocktail (CD11b/Mac-1, 1:800, clone M1/70, #101210; B220, 1:400, clone RA3-6B2, #103210; CD5, 1:400, clone 53-7.3, #100610; Ter119, 1:200, clone TER-119, #116210; Ly-6G/Ly-6C/Gr-1, 1:800, clone RB6-8C5, #108410; CD4, 1:800, clone GK1.5, #100410; CD8a, 1:800, clone 53-6.7, #100710; all coupled to PE-Cy5 and from Biolegend), CD150-APC (1:100, clone TC15-12F12.2, Biolegend, #115910), CD117/c-Kit-APC-eF780 (1:200, clone 2B8, eBioscience, #47-1171-82), Ly-6A/Ly-6E/Sca-1-PB (1:100, clone E13-161.7, Biolegend, #122520), CD16/32-PE-Cy7 (1:400, clone 93, eBioscience, #25-0161-81), CD41-FITC (1:100, clone MWReg30, eBioscience, #11-0411-81), CD105/endoglin-PE (1:100, clone MJ7/18, eBioscience, #12-1051-82), CD48-PE (1:100, clone HM48-1, eBioscience, #12-0481-81) for myeloerythroid progenitors and hematopoietic stem and progenitor cells (HSPC). CD4-PE-Cy5 (1:800, clone GK1.5, Biolegend, #100410) and CD8-FITC (1:800, clone 53-6.7, Miltenyi Biotec, #130-118-468) for T lymphocyte subsets in thymus. Flow cytometry analysis was performed on a FACSCanto II (BD). Fluorescence-activating cell sorting was performed on a FACSAria III (BD). Data analysis was performed using the FlowJo v10.7 software (TreeStar Inc, Ashland, OR, USA).

**Western blot analysis**

Bone marrow and spleen cells were lysed in RIPA lysis buffer (Abcam, Cambridge, UK) containing a complete Mini EDTA-free protease inhibitor cocktail (Sigma-Aldrich). Protein was quantified by Pierce™ BCA Protein Assay Kit (Thermo Fisher Scientific) and equal protein amounts were mixed with 2X Laemmli buffer (1:1) supplemented with 10% β-mercaptoethanol and incubated at 95 °C for 10 min. Proteins were separated on 10% SDS-PAGE gels (Biorad, Munich, Germany) using a Mini-PROTEAN electrophoresis system (BioRad), and transferred to PVDF membranes (Thermo Fisher Scientific). Membranes were blocked for 30 min in 5% nonfat dry milk in TBS-T and were incubated overnight at 4 °C with primary antibodies followed by 1 h incubation with the secondary horseradish peroxidase-conjugated antibody. Immunodetection was performed with UltraScence Western Substrate (Bio-Helix, Keelung, Taiwan) and Amersham Imager 600 (GE Healthcare, Chicago, IL, USA). The following antibodies were used: mouse anti-FLAG M2 antibody (1:1000; F1804; Sigma), and mouse anti-β-actin-Peroxidase (1:20000; A3854; Sigma).

**Overexpression of FLAG-Zrsr2 construct**

Wild-type or mutant Zrsr2 cDNA sequences linked to the FLAG epitope were cloned into the expression vector pcDNA3.1 by VectorBuilder. For vector amplification, recombinant bacteria bearing the wild-type (pcDNA3.1-FLAG-Zrsr2 wt) or mutant (pcDNA3.1-FLAG-Zrsr2 mutant) overexpression vector (VectorBuilder) were grown for 1 hour on shaking at 37°C in 300 µl of LB (Thermo Fisher Scientific) supplemented with 100 μg/ml ampicillin. Then, the culture was seeded in a LB-agar dish with 100 μg/ml ampicillin and incubated overnight at 37°C. Next day, one isolated colony was picked out and grown in 4 ml of LB with 100 μg/ml ampicillin at 37°C under agitation overnight. Plasmidic DNA was extracted using the QIAprep Spin Miniprep kit (QIAGEN) following the manufacturer's instructions. For transfection in HEK293T cells, 15 x 10^4^ cells were seeded in 2 ml of DMEM high glucose (Gibco) supplemented with 10% FBS (Gibco), 100 U/ml penicillin and 100 μg/ml streptomycin (Gibco) in 6-well plates and allowed to grow at 37°C and 5% CO_2_ overnight. Next day, cell medium was renewed and cells were transfected with1 μg of construct using FuGENE reagent (Promega) according to the manufacturer's instructions. After 72 h of growth at 37°C and 5% CO_2_, cells were harvested and proteins were extracted with RIPA (Abcam) for Western blotting.

**PB analysis, cytomorphology and histopathology**

Peripheral blood (PB) was collected from the facial vein of mice into EDTA-coated tubes (Sarstedt, Nümbrecht, Germany). Complete blood cell counts were obtained using an XN-450 automated cell counter (Sysmex, Barcelona, Spain). Morphological analyses were performed on PB smears and BM cytospins stained with May-Grünwald Giemsa solution (Sigma-Aldrich, Munich, Germany).

For histopathological analyses, spleens were fixed in 4% paraformaldehyde and paraffin-embedded using a Leica ASP6025 tissue processor (Leica Biosystems, Wetzlar, Germany). Samples were sectioned using a Leica RM2245 microtome and stained with hematoxylin/eosin (H&E) using a Dako CoverStainer (Agilent, Santa Clara, CA, USA). PB smears, BM cytospins and spleen/liver sections were visualized and analyzed with a Nikon Eclipse Ci (Nikon, Tokyo, Japan) optical microscope.

***In vitro* colony forming assays**

Colony formation (CFU) assays were performed using methylcellulose media (Methocult™ GF M3534 and Methocult™ SF M3436, STEMCELL Technologies, Vancouver, Canada) according to manufacturer’s instructions. For CFU assays, 50,000 unfractionated BM or 100,000 spleen cells were plated in triplicate and colonies were assessed after 7 days for CFU-GM, and after 10 days for BFU-E. For replating assays, colonies were resuspended in IMDM media, counted, and replated (10,000 cells/plate) in GF M3534 media and scored every 7 days.

**Plasma Cytokine Measurement**

Plasma samples were collected by centrifugation of whole blood at 2000 X g for 15 min. For quantification of the cytokine levels in plasma, a commercially available multiplex bead immunoassay based on the Luminex platform (Cat# MCYTOMAG-70K-08, Mouse Cytokine Magnetic Kit, Millipore) was used according to the manufacturer’s instructions. Samples were analyzed in a MAGPIX instrument (Luminex) and results were obtained with the xPONENT software.

**Global DNA methylation**

DNA was isolated from hematopoietic cells using the QIAmp DNA Mini kit (QIAGEN). Global DNA methylation was assessed by Infinium Mouse Methylation Bead Chip (285K) by Illumina. Data analysis was performed by Diagenode with the Methylation module (version 1.9.0) of the GenomeStudio software (Illumina; version 2011.1). The methylation score for each CpG site was represented as a beta value according to the fluorescent intensity ratio. Background subtraction was conducted with the GenomeStudio software using built-in negative control bead types on the array. Raw data were normalized using the GenomeStudio internal control normalization method for the Illumina 285K assay. Differential DNA methylation positions were identified using the GenomeStudio methylation module. DNA methylation data have been deposited in the ArrayExpress database at EMBL-EBI (www.ebi.ac.uk/arrayexpress) under accession number E-MTAB-11482.

**RT-PCR for the detection of alternative splicing events**

Total RNA was extracted from sorted LSK cells (50,000 per sample) with an RNEasy Micro kit (QIAGEN) followed by on-column DNAse treatment. cDNA was obtained by retrotranscription with oligo(dT) and TaqMan Reverse Transcription Reagents (Thermo Fisher Scientific) in a 20 µl final volume. RT-PCR was performed to validate alternative splicing events previously detected by RNA-seq. Specific primers were designed for this purpose (**Table Supplementary 1**), and PCR reactions were performed with PCR Master Mix 2X (Thermo Fisher Scientific) using 40-45 cycles. All PCR products were analyzed on a QIAxcel capillary electrophoresis system (QIAGEN, Hilden, Germany).

**Statistical analysis**

Normality was checked by Shapiro-Wilk and Kolmogorov-Smirnov tests. Equality of variances was checked and assumed equal for all datasets. Ordinary ANOVA was used for comparison among 3-4 groups using Dunnett’s as a post hoc test. Unpaired two-tailed Student’s t-test was used when comparing 2 groups. Otherwise, the non-parametric Kruskal-Wallis test and Dunn’s as a post hoc test were used. All graphs show the mean with all individual data points. Data are presented as mean ± SEM. All analyses were performed with GraphPad Prism version 9 (GraphPad Software, San Diego, CA, USA). Statistical significance was set at *p*-value < 0.05.

**Supplementary Table 1. Primers used in this study.** Primer sequences and application are indicated.

| **Gene** | **Forward** | **Reverse** | **Application** |
| --- | --- | --- | --- |
| ***Zrsr2*** | 5′-AAGGGAAGAAGACTGTCAAGCAGC-3′ | 5′-AAGGGTACCACTACATTGGCT-3′ | Genotyping/Sanger |
| ***Tet2*** | 5′-CTCTCAAGTCACAGAAACACGTG-3′ | 5′-GTTGCTTGAGGCTCTGCAGCTC-3′ | Genotyping/Sanger |
| ***Zrsr2*** | 5′-CAAAACGGGAGCTTGCAGAT-3′ | 5′-TCTAGGCTTGAGTCAGGGTC-3′ | RT-qPCR |
| ***Hprt*** | 5′-GCCCTTGACTATAATGAG-3′ | 5′-GATAAGCGACAATCTACC-3′ | RT-qPCR |
| ***Zrsr2*** | 5′-TAAGGTCAGCTGCAACCTGG-3′ | 5′-CCCTGTGTCTGCTACTGCTC-3′ | Validation RT-PCR |
| ***Dusp1*** | 5′-GAAGCGTTTTCGGCTTCCTG-3′ | 5′-TTCCGAGAAGCGTGATAGGC-3′ | Validation RT-PCR |
| ***Tgfbr2*** | 5′-GGTCTATGACGAGCGACGG-3′ | 5′-GTGGACAGTCTCACATCGCA-3′ | Validation RT-PCR |
| ***Fgf11*** | 5′-AAGCTGGGTCACTACATGGC-3′ | 5′-AGAGAAGGCTCCCGGTACAT-3′ | Validation RT-PCR |
| ***Per1*** | 5′-AAAGTCCTTCCCTGCCAGTC-3′ | 5′-CTCTGAAGCGGTTGAGGAAG-3′ | Validation RT-PCR |
| ***Ttbk2*** | 5′-ACCCTGAGCTAGACCCTCGT-3′ | 5′-TCATTGCAAAACCCTGATGA-3′ | Validation RT-PCR |
| ***Frrs1*** | 5′-CATCACCCTTCCTGAGGCTA-3′ | 5′-ACCAGGACTCCAATGCTGAC-3′ | Validation RT-PCR |

**Supplementary Table 4. Comparison between transcriptomic and epigenomic analyses.** **a)** List of genes present in the intersection of the Venn diagram representing significantly differentially expressed/methylated genes (related to Supplementary Fig. 5). **b)** DNA methylation parameters for three selected differentially methylated genes (related to Supplementary Fig. 5).

a

| Sgk1 | Tcf7l1 | **Tgfbr3** | Tgfbr3l | Trrap |
| --- | --- | --- | --- | --- |
| Hspa1b | Lrp1 | Sertad2 | Dpp8 | Dip2c |
| Pygm | Vamp1 | Tiam2 | Rinl |  |
| Zfp503 | Chd7 | Plod3 | Amigo2 |  |
| Prkd2 | D2hgdh | Ablim1 | Pik3cg |  |
| Gpc3 | Mmp2 | Txn1 | Ntng1 |  |
| Cacnb1 | Syp | Osm | Usp48 |  |
| Osgin1 | Dpp4 | Lrch2 | Gm5148 |  |
| Map3k6 | Cdk5rap1 | Nod2 | Gcnt2 |  |
| Dusp6 | Rasa4 | Nuak2 | Unc5a |  |
| Mcf2 | Fabp4 | Wdhd1 | Vps13d |  |
| Hpse | Ogt | Unc93b1 | Iqsec2 |  |
| Cd33 | Smox | Lonrf3 | Fbxl19 |  |
| Bhlhe40 | Scn1b | Pik3r3 | Gabpb1 |  |
| **Blnk** | Akt3 | Nyap1 | Smarca2 |  |
| Hemgn | **Mpo** | Zfyve27 | Apc2 |  |
| Sall2 | Haghl | Il16 | 0610010K14Rik | |
| Gas2 | Stxbp4 | Adamts1 | BC016579 |  |
| BC048403 | Zfhx2 | Lss | Zscan10 |  |
| Mcee | Rhoq | Timm17b | Pparg |  |
| Klhl4 | Rab44 | Nhsl2 | Dnah12 |  |

b

|  | **β-values WT average** | **β-values *Zrsr2^m/m^Tet2^−/−^* average** | ***Zrsr2^m/m^Tet2^−/−^* DiffScore** | ***Zrsr2^m/m^Tet2^−/−^***  **Delta β-values** |
| --- | --- | --- | --- | --- |
| **Blnk** | 0,5675 | 0,3380 | -58,0989 | -0,2295 |
| **Mpo** | 0,5041 | 0,6401 | 16,0003 | 0,1359 |
| **Tgfbr3** | 0,6986 | 0,8152 | 19,4350 | 0,1166 |


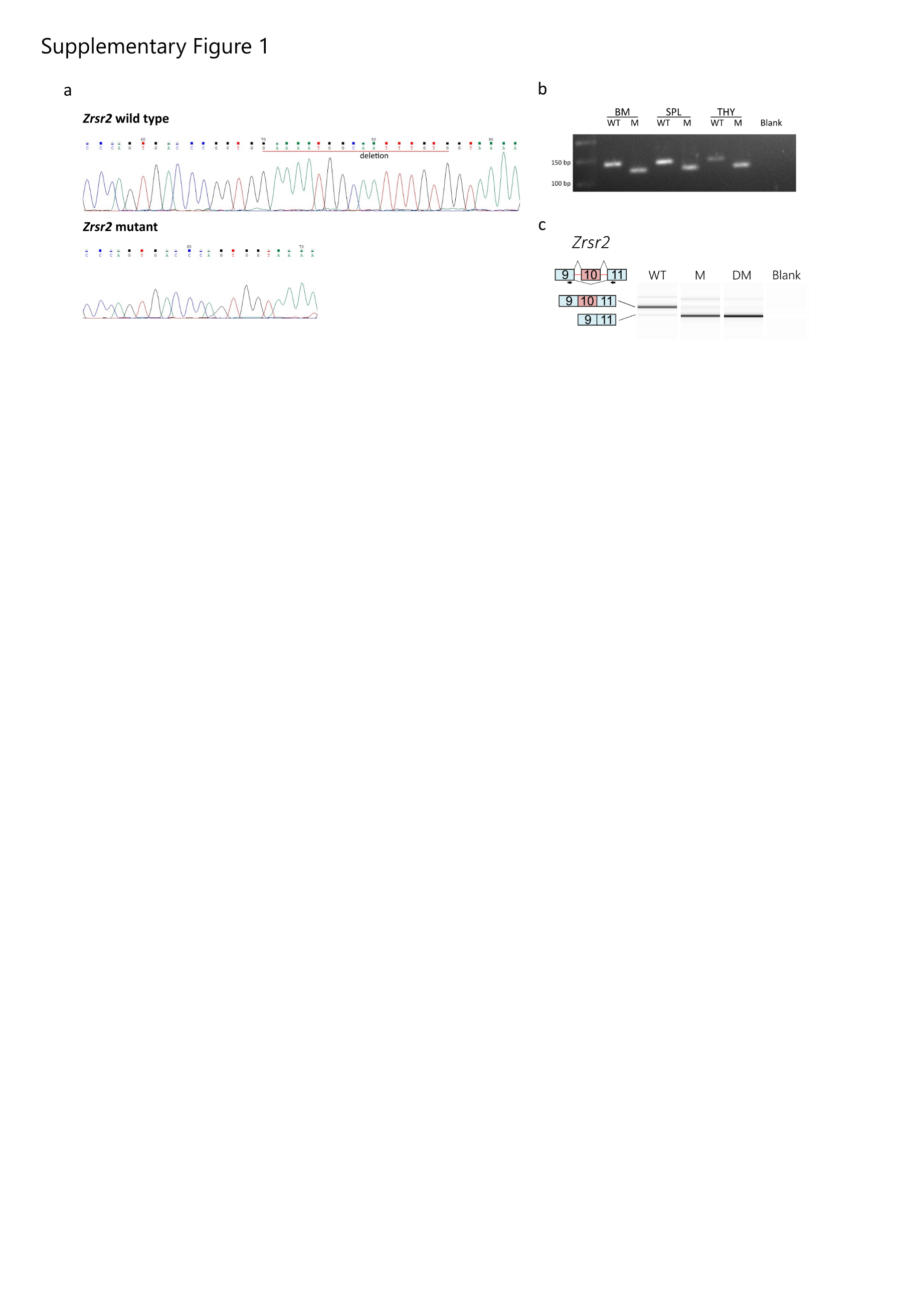


**Supplementary Figure 1. Generation of a germline *Zrsr2* allele. a)** Sanger sequencing chromatograms in WT and *Zrsr2^m/m^* genomic DNA. Seventeen-nucleotide deletion is indicated in red. **b)** PCR using genomic DNA from hematopoietic tissues (BM: bone marrow; SPL: spleen; THY: thymus). **c)** RT-PCR of *Zrsr2* in LSK cells sorted from WT, *Zrsr2^m/m^* (M), and *Zrsr2^m/m^Tet2*^−/−^ (DM) mice. WT (n = 1), M (n = 2), DM (n = 4). Bands corresponding to WT and mutant transcripts are depicted. *Zrsr2* mutant mRNA presented an exon 10 skipping in addition to the 17-nucleotide deletion.


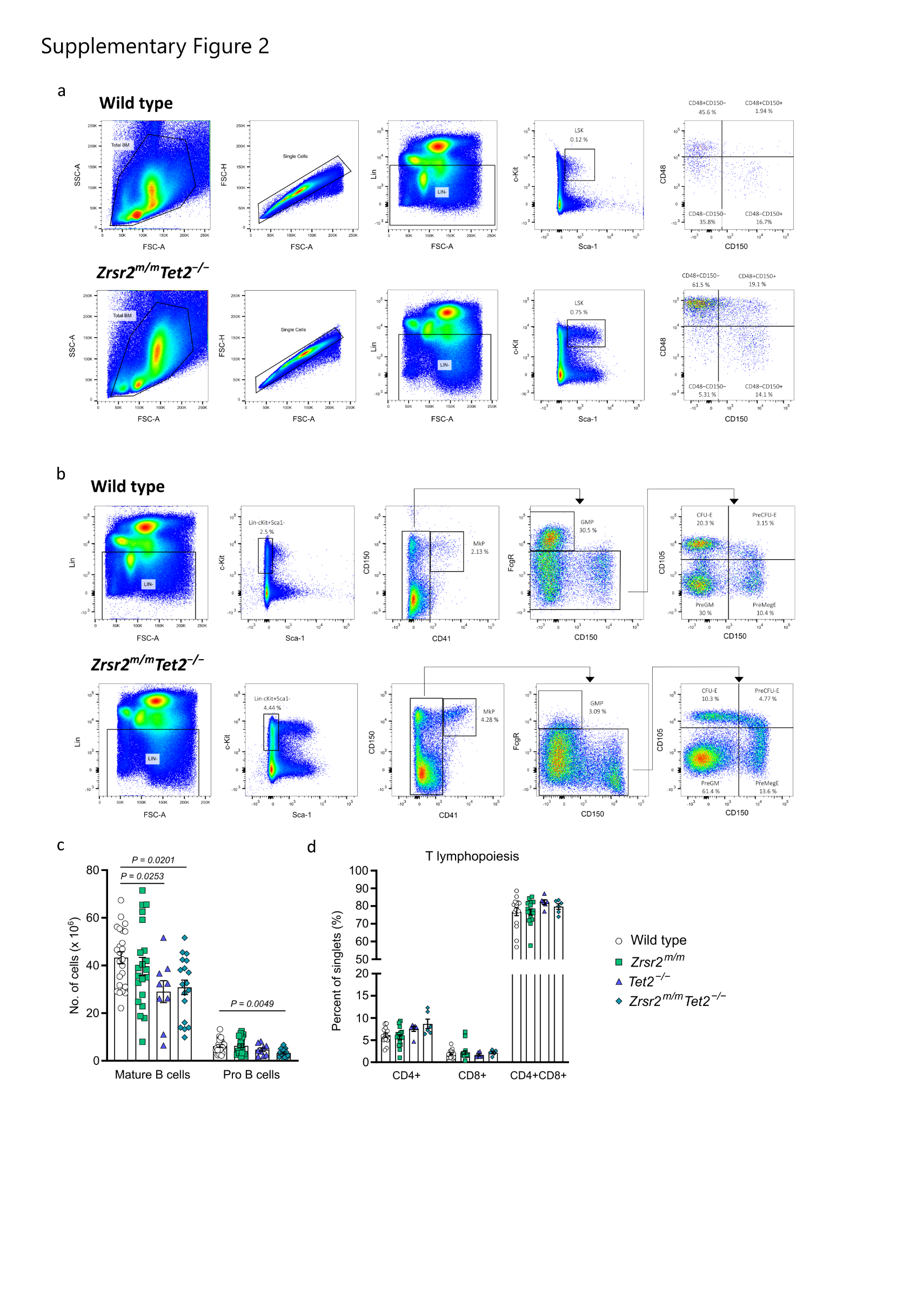


**Supplementary Figure 2. FACS analysis of HSC, myelo-erythroid and B progenitors, and T lymphocytes. a)** Representative FACS plots of LSK compartment in WT and *Zrsr2^m/m^Tet2^−/−^* BM cells. LSK as percentage of total cells. SLAM subpopulations as frequency of LSK cells. **b)** Representative FACS plots and gating strategy for myelo-erythroid progenitors in BM. Progenitors as percentage of total cells. Myelo-erythroid progenitors as frequency of progenitors. **c)** Counts of mature B cells (CD19^+^B220^+^) and Pro-B progenitors (CD19^+^B220^+^CD43^+^) in BM of 3-months old WT (n = 24), *Zrsr2^m/m^* (n = 21), *Tet2*^−/−^ (n = 9), and *Zrsr2^m/m^Tet2*^−/−^ (n = 18) mice. **d)** Frequency of T lymphocyte subsets in thymus from 3-months-old WT (n = 16), *Zrsr2^m/m^* (n = 17), *Tet2*^−/−^ (n = 6), and *Zrsr2^m/m^Tet2*^−/−^ (n = 6) mice. Data represent mean ± SEM.


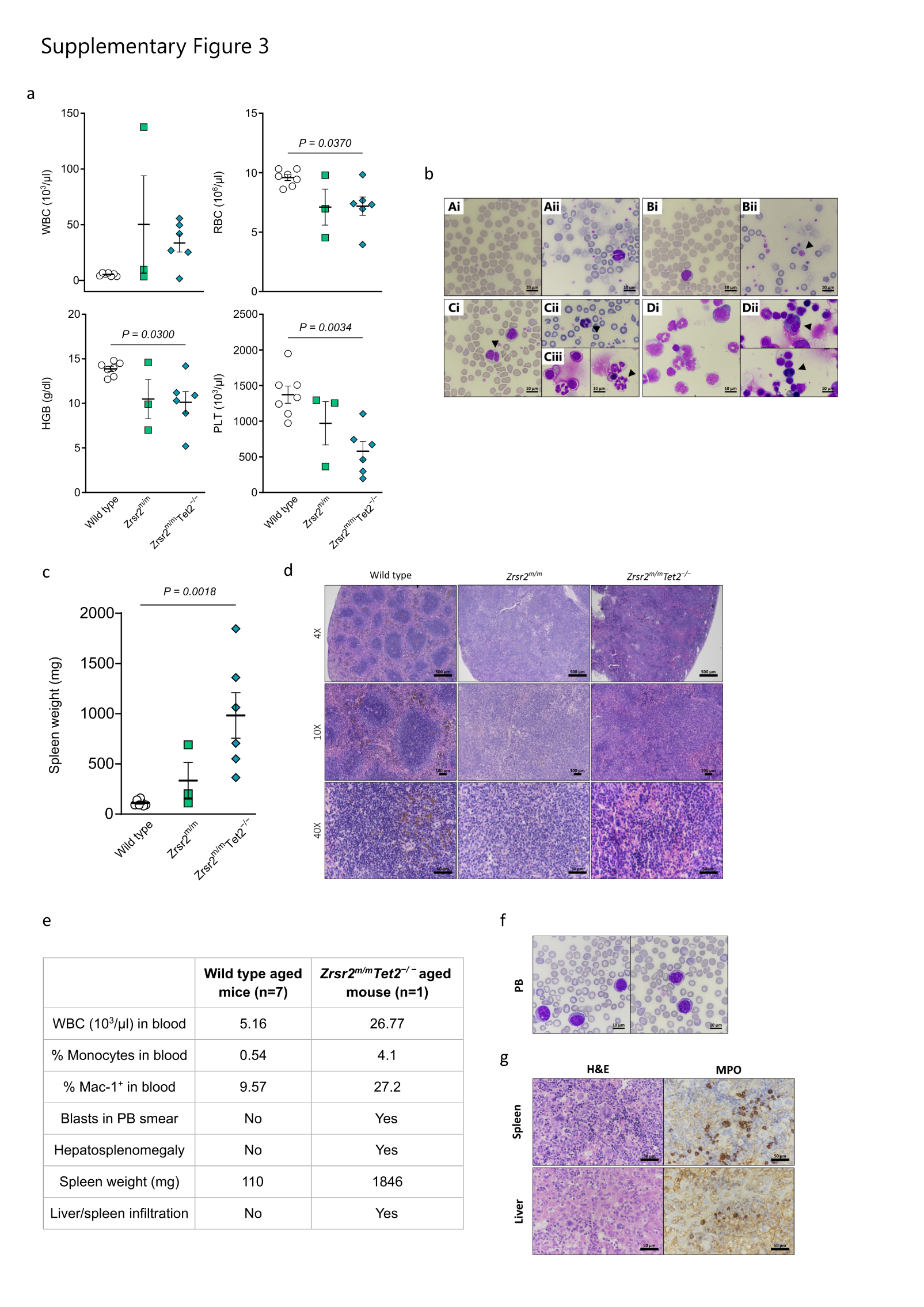


**Supplementary Figure 3. Hematological phenotype of aged control (24 months), Zrsr2 mutant (24 months) and *Zrsr2^m/m^Tet2****^−/−^* **mice (18 months).** **a)** Hematological parameters from WT (n = 7), *Zrsr2^m/m^* (n = 3), and *Zrsr2^m/m^Tet2^−/−^* mice (n = 6). WBC: white blood cells, RBC: red blood cells, HGB: hemoglobin, PLT: platelets. **b)** Normal RBC from WT (Ai) and dysplastic RBC from *Zrsr2^m/m^Tet2^−/−^* (Aii), showing RBC anisocytosis. Normal platelets from WT (Bi) and enlarged platelets from *Zrsr2^m/m^Tet2^−/−^* mice (Bii). Hyposegmented granulocytes (Ci), granulocytes with nuclear fragmentation (Cii), and hypersegmented neutrophils (Ciii) from *Zrsr2*^m/m^*Tet2*^−/−^ mice. Normal progenitors from WT (Di) and binucleated erythroid precursors from *Zrsr2*^m/m^*Tet2*^−/−^ mice (Dii). **c)** Spleen weight from WT (n = 7), *Zrsr2^m/m^* (n = 3), and *Zrsr2^m/m^Tet2^−/−^* (n = 6) mice. **d)** Spleen sections from WT (n = 7), *Zrsr2^m/m^* (n = 3), and *Zrsr2^m/m^Tet2^−/−^* (n = 3) mice stained with H&E. **e)** Characteristics of an aged *Zrsr2^m/m^Tet2^−/−^* mouse suggestive of progression to secondary AML. **f)** PB smears of an aged *Zrsr2^m/m^Tet2^−/−^* mouse consistent with sAML. A high proportion of immature, rounded-shaped blasts with alike morphology was observed. **g)** Spleen/Liver sections showing H&E and myeloperoxidase (MPO) staining of an aged *Zrsr2^m/m^Tet2^−/−^* mouse with sAML. Spleen and liver histology appeared altered, with infiltration of hematopoietic cells many of which were MPO positive.


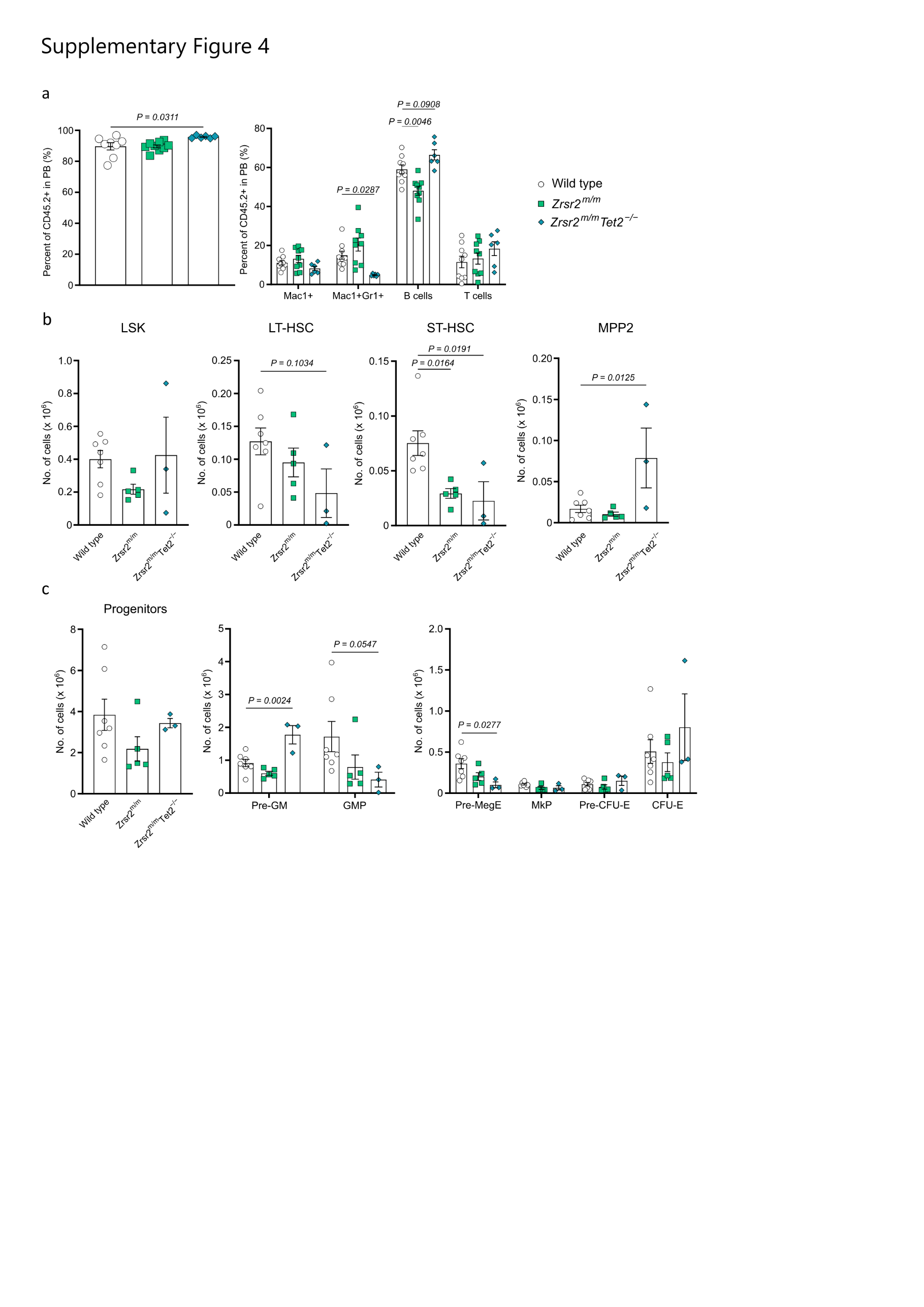


**Supplementary Figure 4. Combined *Zrsr2-Tet2* mutations in HSPC enhance BM repopulation capacity and cause cell-autonomous hematopoietic alterations.** **a)** Donor (CD45.2^+^) chimerism in peripheral blood at 8-12 weeks post-transplantation. The contribution to B cells (B220), T cells (CD4/CD8), neutrophilic granulocytes (Mac1^+^Gr1^+^), and other monocytic/granulocytic cells (Mac1^+^) was analyzed. WT (n = 9), *Zrsr2^m/m^* (n = 9), and *Zrsr2^m/m^Tet2^−/−^* (n = 6). **b)** Absolut count of donor-derived LSK cells and LSK subpopulations. WT (n = 7), *Zrsr2^m/m^* (n = 5), and *Zrsr2^m/m^Tet2^−/−^* (n = 3). Mice were analyzed at 15 months post-transplantation. **c)** Absolut count of donor-derived progenitors and of donor-derived myelo-erythroid progenitors. WT (n = 7), *Zrsr2^m/m^* (n = 5), and *Zrsr2^m/m^Tet2^−/−^* (n = 3). Mice were analyzed at 15 months post-transplantation.


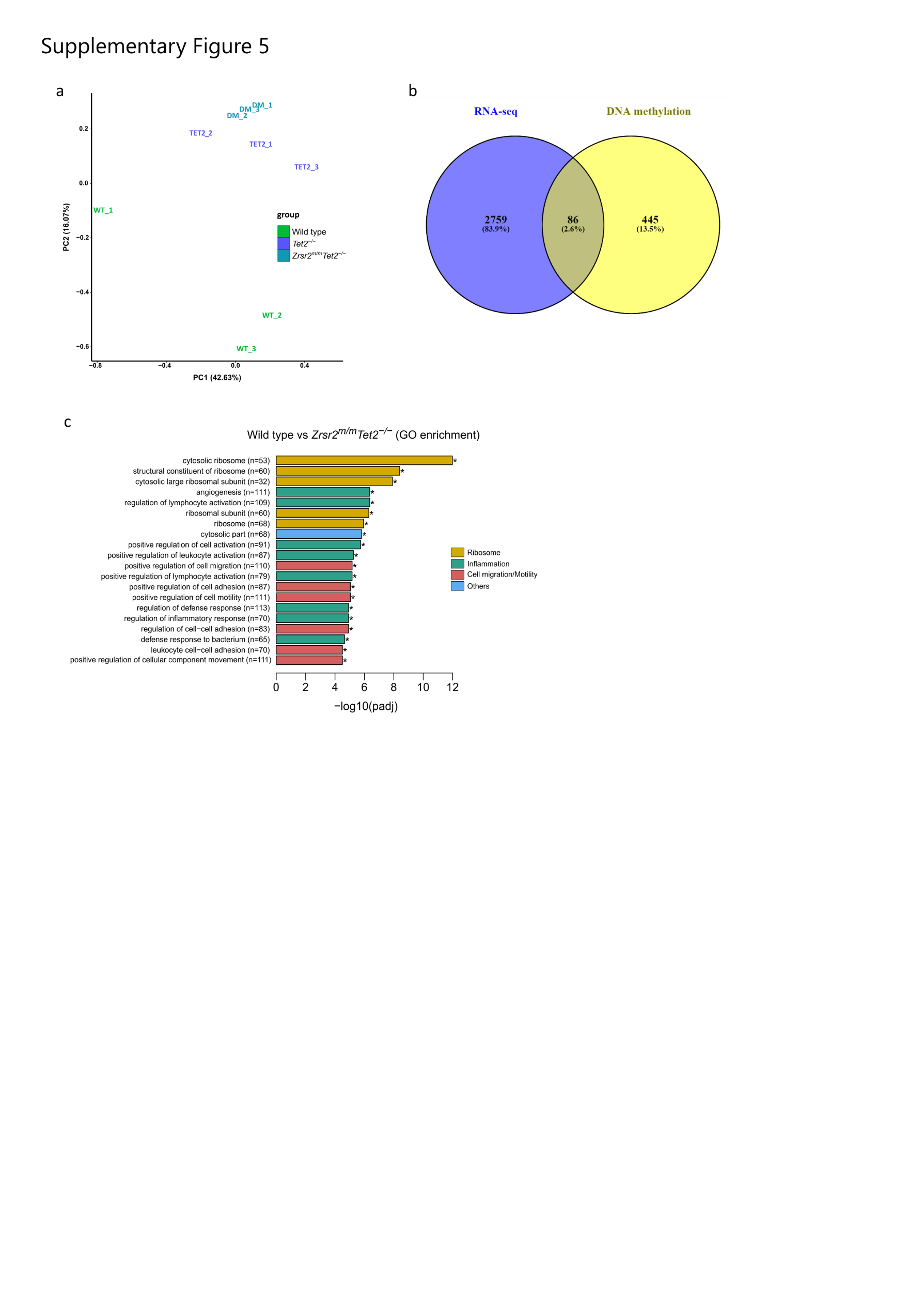


**Supplementary Figure 5. Analysis and comparison between transcriptome and epigenetic analysis.** **a)** Principal component analysis (PCA) of WT, *Tet2^−/−^*, and *Zrsr2^m/m^Tet2^−/−^* samples showing CpG methylation levels. n = 3 replicates/genotype. **b)** Venn diagram showing the overlap of dysregulated genes from gene expression and DNA methylation analysis in *Zrsr2^m/m^Tet2^−/−^* mice. **c)** Gene ontology (GO) enrichment analysis of DEG in LSK from WT vs *Zrsr2^m/m^Tet2^−/−^* mice.

**
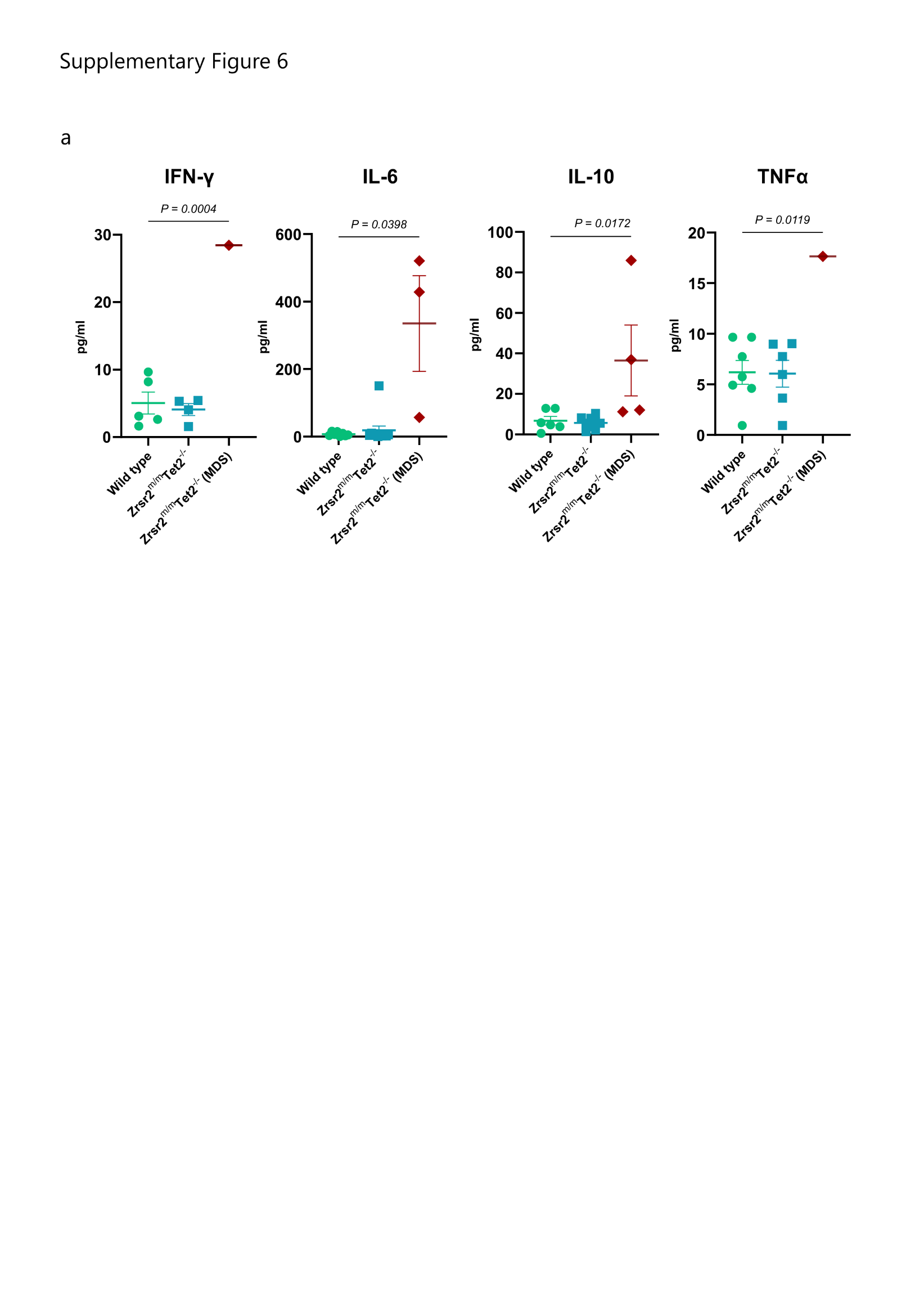
**

**Supplementary Figure 6. Levels of pro-inflammatory cytokines.** **a)** Plasma cytokine levels in 3-months-old WT, *Zrsr2^m/m^Tet2^−/−^*, and *Zrsr2^m/m^Tet2^−/−^* mice displaying MDS determined by bead-based Luminex xMAP technology.


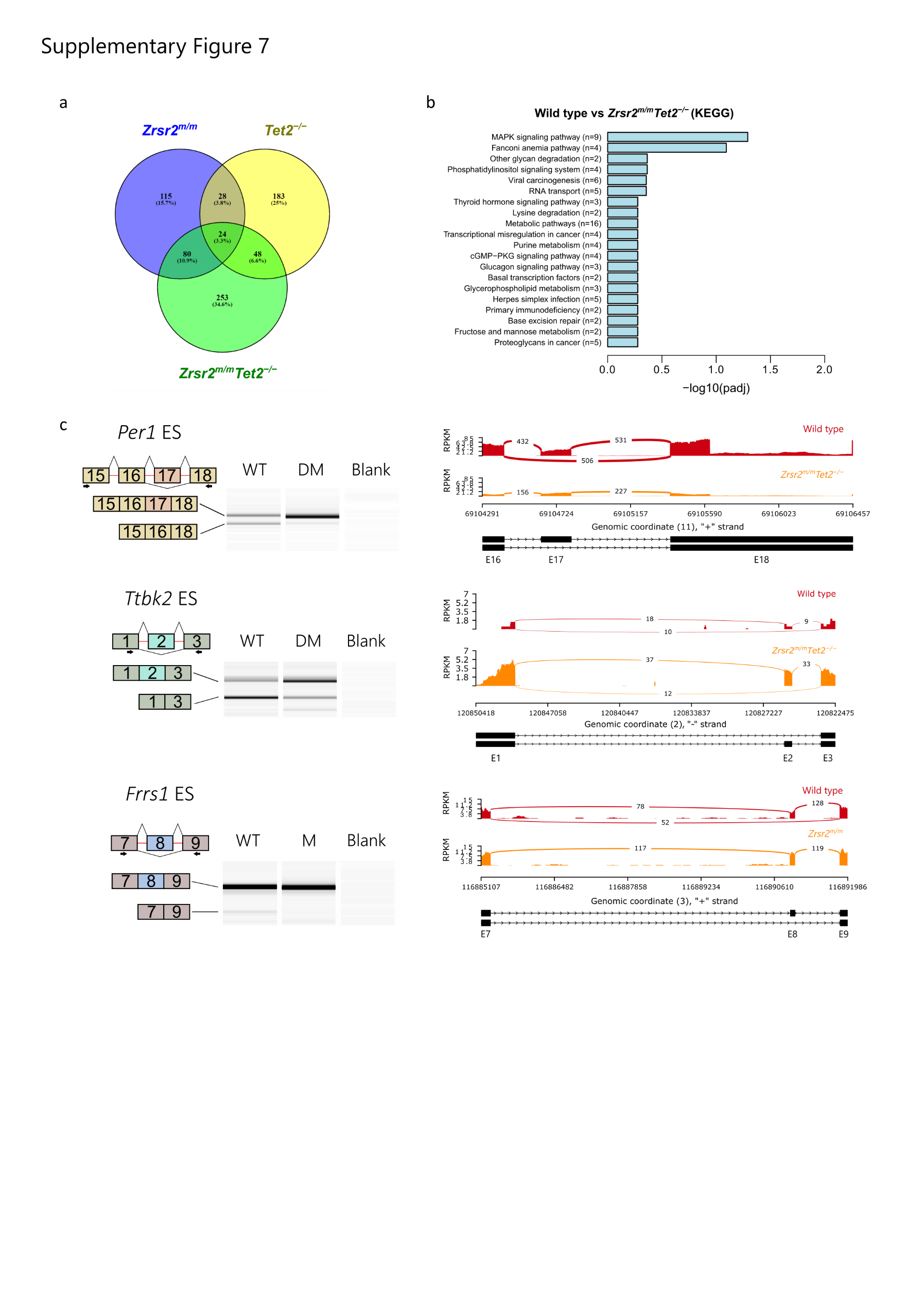


**Supplementary Figure 7. Analysis of aberrant mRNA splicing events identified by RNA-seq. a)** Venn diagram showing the overlap of alternatively spliced genes among the different comparison groups (*Zrsr2^m/m^* vs WT, *Tet2*^−/−^ vs WT, *Zrsr2^m/m^Tet2^−/−^* vs WT). **b)** KEGG enrichment analysis of alternatively spliced genes in LSK from *Zrsr2^m/m^Tet2^−/−^* vs WT. **c)** RT- PCR in LSK cells and Sashimi plots showing exon skipping events in *Per1*, *Ttbk2*, and *Frrs1* transcripts. WT: wild-type; DM: double mutant *Zrsr2^m/m^Tet2^−/−^*; M: mutant *Zrsr2^m/m^*.

**Supplementary References**

1. Gómez-Redondo I, Ramos-Ibeas P, Pericuesta E, Fernández-González R, Laguna-Barraza R, Gutiérrez-Adán A. Minor Splicing Factors Zrsr1 and Zrsr2 Are Essential for Early Embryo Development and 2-Cell-Like Conversion. Int J Mol Sci. 2020; 21:4115.

2. Ramos-Ibeas P, Calle A, Fernández-González R, Laguna-Barraza R, Pericuesta E, Calero A et al. Intracytoplasmic sperm injection using DNA-fragmented sperm in mice negatively affects embryo-derived embryonic stem cells, reduces the fertility of male offspring and induces heritable changes in epialleles. PLoS One. 2014; 9:e95625.
